# Dynamic Regulation of Level Set Parameters Using 3D Convolutional Neural Network for Liver Tumor Segmentation

**DOI:** 10.1155/2019/4321645

**Published:** 2019-02-24

**Authors:** Zhuofu Deng, Qingzhe Guo, Zhiliang Zhu

**Affiliations:** College of Software, Northeastern University, Shenyang 110004, China

## Abstract

Segmentation of liver tumors plays an important role in the choice of therapeutic strategies for liver disease and treatment monitoring. In this paper, we generalize the process of a level set with a novel algorithm of dynamic regulation to energy functional parameters. The presented method is fully automatic once the tumor has been detected. First, a 3D convolutional neural network with dense layers for classification is used to estimate current contour location relative to the tumor boundary. Second, the output 3D CNN probabilities can dynamically regulate parameters of the level set functional over the process of segmentation. Finally, for full automation, appropriate initializations and local window size are generated based on the current contour position probabilities. We demonstrate the proposed method on the dataset of MICCAI 2017 LiTS Challenge and 3DIRCADb that include low contrast and heterogeneous tumors as well as noisy images. To illustrate the strength of our method, we evaluated it against the state-of-the-art methods. Compared with the level set framework with fixed parameters, our method performed better significantly with an average DICE improvement of 0.15. We also analyzed a challenging dataset 3DIRCADb of tumors and obtained a competitive DICE of 0.85 ± 0.06 with the proposed method.

## 1. Introduction

The segmentation of liver tumors in computed tomography (CT) is required for assessment on tumor load, prevention of liver diseases, treatment planning, prognosis, and monitoring of treatment response [1, 2]. Heterogeneity in the shape and texture of the liver and visible tumors in CT scans are significant biomarkers for diseases progression [3]. In clinical routine, manual techniques are applied, but cost and amount of tedious time are unacceptable [4]. Therefore, it is highly desirable to have approaches that can automatically perform segmentation. However, there are some challenges in automatic algorithms such as low contrast between tumors and liver, different levels of contrast, inhomogeneous shapes, and locations of tumors. Thus, accurate delineation of malignant tissue by computer assistance remains difficult.

Several state-of-the-art algorithms, including thresholding, region-based method, active contour models, graph cut, and machine learning, have been introduced to solve this problem [5]. For instance, Moltz et al. combined a threshold-based idea with model-based morphological processing adapted to liver tumor segmentation [6]. Cao et al. proposed a simple and fast method based on iterative relative fuzzy connectedness (IRFC) derived from region growing [7]. Yan et al. used a marker-controlled watershed transformation to segment 3D liver metastases in volumetric CT images [8]. These methods are efficient but too sensitive to noise and incompetent for delineation of complex shape or texture of objects. Recently, active contour based on curve evolution is popular because of its adaptiveness to complex shapes [9]. Some models, like SNAKE [10], transform curve motion into discrete points', which leads to a poor segmentation especially in the narrow or sharp region. To overcome these difficulties, the level set is proposed and considered on a higher level dimension. It has the ability of handling image noise, intensity heterogeneity, and discontinuous object boundaries. Level set methods include models based on edge [11–13] or region [14–16]. Edge-based ones are good at dealing with irregular boundaries and low contrast texture. Region-based ones study spatial statistics of areas but pay not enough attentions for details. Lankton and Tannenbaum gave a novel algorithm relying on the local point region to build an energy model which reflects specific features of the object and serves the confused condition properly [17]. Li et al. introduced another hybrid model integrating edge and region models. These hybrid models are more competitive and preferred by most researchers, providing a more credible segmentation.

In level set works, the choice of energy functional parameters causes troubles. Inappropriate parameters result in disabled automatic segmentation [18]. Lankton et al. argued that parameters have close relationships with direction and speed of curve evolution, and fixed ones limit performance of level set segmentation. Even though some researches tried to test the selected value over a series of the training set for the entire dataset, it is hard to maximize the effect of segmentation on all of them. Choice by a trial is time-consuming and laborious. At present, this work entirely depends on expert experience. Some papers attempted to estimate them. Li et al. [18] introduced a method to adjust parameters according to classification of pixels in region of interest (ROI). Unfortunately, the value is initialized once at the beginning of work and remains constant. Oliviera et al. [19] provided a mechanism to evaluate parameters from analysis of the dataset. But for a specific image, the selected parameter cannot run primarily. Baillard et al. [20] devised a solution to take full considerations for every point position. Over steps, a point on the contour at first determines its status. If it belongs to the foreground, it locally extends outwards. And if not, it moves in the opposite direction. This classification depends on Gaussian and the shifted Rayleigh statistical distribution models via the training set by maximizing the posterior probability. However, because of the limited effective classifier for tumors in medical imaging, its prediction accuracy would be doubted. Hoogi et al. [21] predicted the position of evolving contour using CNN in the first time. By adaptive parameters, the zero level set is able to escape from local minima acquiring better results. However, it lacks the research on 3D segmentation that reflects more significance in practice.

Furthermore, deep learning techniques have appeared as a powerful alternative for supervised learning applications like classification, detection, and segmentation. Convolutional neural network (CNN) [22, 23] can learn highly discriminative features and have been widespread with predominance on a variety of problems including medical imaging. Ciresan et al. [24] adopt classical CNN for the segmentation of the neural membrane. Obviously, this strategy has two drawbacks. First, it costs too much computing time since the network runs separately for each pixel, and then there is a lot of redundancy due to overlapping patches. In addition, there is a trade-off between localization accuracy and the use of context. For more accurate localization, Ronneberger et al. [25] gave a more elegant architecture U-Net derived from the fully convolutional network (FCN) [26], which consists of a contracting path to capture context and a symmetric expanding path. Although the algorithm gains a certain achievement, the result of the object specifically in edge is too coarse to be accepted for medical image segmentation. Kamnitsas et al. [27] incorporated conditional random field (CRF) into FCN. Details of segmentation are refined by the fuzzy classification based on features of intensity and pixel position. To deal with the variations of tumor shape, size, intensity, and texture, Chlebus et al. [28] proposed an automatic liver tumor segmentation with random forest-based candidate filtering that was built on the U-Net. Similarly, Han [29] presented another algorithm derived from U-Net, which combined the ResNet model as a part of baseline. Both of them are 2D networks that pay little attention to features of adjacent slices and provide lower performance in 3D segmentation. Christ et al. [30] used cascaded fully convolutional neural networks, enabling the 3D segmentation for low-contrast CT images. The first pipeline extracted the liver as volume of interest (VOI) input, serving for the tumor segmentation via the second network of FCN. As a result of the limited separate FCN's ability, the reported evaluations were not satisfied. Moghbel et al. gave a fully automatic segmentation without any interactions, integrating cuckoo optimization and fuzzy c-means with random walkers. Foruzan and Chen [31] and Wu et al. [32] presented semiautomatic methods depending on watershed and graph cut, and they obtained improved results better than previous ones. The algorithm proposed by Huang et al. [33] also relied on an accurate eclipse initialization enclosing the tumor. Because these semiautomatic methods require prior knowledge of accurate initialization, and a comparable fully automatic segmentation is necessary in future.

In this paper, we propose a significant improvement on the traditional level set process, a novel algorithm of dynamic regulation to functional parameters over iterations using the 3D convolutional neural network (DRLS). Our method is a multistage process. A 3D convolutional neural network is designed and trained for identifying the current contour (actually a volume in the 3D level set) position. Its output probabilities were used to estimate various parameters during the process of the level set. At first, for fully automatic segmentation, an appropriate initialization is given by 3D CNN which helps to select an initial contour close to tumor boundary relatively. In addition, compared with the level set with fixed functional parameters, the proposed method can regulate parameters dynamically based on contour location information. Local window size plays a crucial role in zero level set evolution. In our work, a proper size is reestimated in each iteration. If it is far away from the tumor boundary, the window size should be enlarged comparatively. When combined with estimation of level set multiparameters, we supply a generalization of the segmentation process, applying the same energy model of the popular level set framework and deep learning skills for any given dataset. Our method requires only a proper VOI to produce candidate initializations. There is no need of a more accurate initial contour manually with expertise. Contrary to current level set frameworks, the proposed method has little sensitivity to accurate initialization and does not include any assumptions about tumor characteristics. Consequently, its performance is better in highly diverse datasets that include low contrast, noisy, and heterogeneous tumors. Experiment results demonstrated that the method runs stable and competitive to state-of-the-art methods, which showed the strength of ours.

To the best of our knowledge, this is the first use of 3D CNN to join in the level set for 3D segmentation. Through dynamic parameters, the proposed hybrid method of the 3D level set and deep learning is able to resolve the problems of low contrast, abnormal shape, and variant features of texture tumors, resulting in a generalized segmentation solution than methods available to date.

The rest of this paper is organized as follows.  Section 2 introduces the details of the proposed method. Sections 3 and 4 present the experimental results and related discussions. Finally, the conclusion is summarized in Section 5.

## 2. Materials and Methods

### 2.1. Energy Models

#### 2.1.1. Uniform Modeling (UM) Energy

In the development history of the active contour method, lots of energy models were proposed for special application. A well-known example of an energy that uses a constant intensity model is the Chan–Vese energy [14], which we will refer to as the uniform modeling energy:

(1) $$\begin{array}{c} E_{\text{UM}}=\int_{\Omega_{y}}H_{\phi \left(y\right)}{\left(I\left(y\right)-u\right)}^{2}+\left(1-H_{\phi \left(y\right)}\right){\left(I\left(y\right)-v\right)}^{2}dy, \end{array}$$

(2) $$\begin{array}{c} F_{\text{UM}}=\lambda_{1}H_{\phi \left(y\right)}{\left(I\left(y\right)-u_{x}\right)}^{2}+\lambda_{2}\left(1-H_{\phi \left(y\right)}\right){\left(I\left(y\right)-v_{x}\right)}^{2}dy. \end{array}$$

This energy equation (1) models the foreground and background as constant intensities represented by their means, *u* and *v*. By replacing *u* and *v* with corresponding *u*_*x*_ and *v*_*x*_ as equation (2), we obtain the speed iterative formula of the local version UM model written as

(3) $$\begin{array}{c} \frac{\partial \phi }{\partial t}\left(x\right)=\delta \phi \left(x\right)\int_{\Omega_{y}}B\left(x,y\right)\left(\lambda_{1}{\left(I\left(y\right)-u_{x}\right)}^{2}-\lambda_{2}{\left(I\left(y\right)-v_{x}\right)}^{2}\right)+\mu \delta \phi \left(x\right)\text{div}\left(\frac{▽\phi \left(x\right)}{\left|▽\phi \left(x\right)\right|}\right), \end{array}$$

where *u*_*x*_ and *v*_*x*_ are mean intensities of point's local region. The minimum is obtained when each point on the curve has moved such that the local interior and exterior about every point along the curve is best approximated. *λ*_1_ and *λ*_2_ control two subregions energies trade-off.

#### 2.1.2. Mean Separation (MS) Energy

Another important global region-based energy that relies on mean intensities is proposed by Yezzi et al. [34], which is determined by the discrepancy between foreground and background, referred to as mean separate (MS) energy:

(4) $$\begin{array}{c} E_{\text{MS}}=\int_{\Omega_{y}}{\left(u-v\right)}^{2}. \end{array}$$

This energy has the important basis that foreground and background regions should have maximally separate mean intensities. Minimizing the energy causes the curve to evolve so that interior and exterior means have the largest difference possible. *u* and *v* are mean intensity of foreground and background in the image. MS model encounters difficulties in complex texture like the medical image since the energy functional tends to be trapped in local minima. Lankton et al. came up with a local model, converting Yezzi's model into point's local region on the curve [17]:

(5) $$\begin{array}{c} \frac{\partial \phi }{\partial t}\left(x\right)=\delta \phi \left(x\right)\int_{\Omega_{y}}B\left(x,y\right)\cdot {▽}_{\phi \left(y\right)}F\left(I\left(y\right),\phi \left(y\right)\right)dy+\mu \delta \phi \left(x\right)\text{div}\left(\frac{▽\phi \left(x\right)}{\left|▽\phi \left(x\right)\right|}\right), \end{array}$$

(6) $$\begin{array}{c} F_{\text{MS}}=-\left(\lambda_{1}u_{x}-\lambda_{2}v_{x}\right)\cdot \left(\frac{\lambda_{1}\left(I\left(x\right)-u_{x}\right)}{A_{\text{in}}}+\frac{\lambda_{2}\left(I\left(x\right)-v_{x}\right)}{A_{\text{out}}}\right). \end{array}$$

Equation (5) is the speed iterative formula of Lankton's standard deduction. *B*(*x*, *y*) represents the local region of a point on the contour, like a rectangular or circle. The function *F* gives a generic internal energy measure used to describe local adherence to a given model. If MS model is chosen, *F* could be replaced with *F*_MS_ equation (6), where *A*_in_ and *A*_out_ are areas about interior and exterior, respectively. Meanwhile, *λ*_1_ and *λ*_2_ control the magnitude of two forces in opposite directions. In order to keep the curve smooth as possible, a regularization term is added. For the 3D level set, *B*(*x*, *y*, *z*) and *F*(*x*, *y*, *z*) join instead of the previous relative ones. Equation (7) is the result by inserting equation (6) to equation (5):

(7) $$\begin{array}{c} \frac{\partial \phi }{\partial t}\left(x\right)=\delta \phi \left(x\right)\int_{\Omega_{y}}B\left(x,y\right)\left(\frac{\lambda_{1}{\left(I\left(y\right)-u_{x}\right)}^{2}}{A_{u}}-\frac{\lambda_{2}{\left(I\left(y\right)-v_{x}\right)}^{2}}{A_{v}}\right)+\mu \delta \phi \left(x\right)\text{div}\left(\frac{▽\phi \left(x\right)}{\left|▽\phi \left(x\right)\right|}\right). \end{array}$$

In practice, the local MS model has a better performance than the UM model in medical image segmentation especially for irregular shape and texture features. To evaluate our proposed method, all datasets were tested on the MS energy model.

### 2.2. The Proposed Method

**Figure 1:**
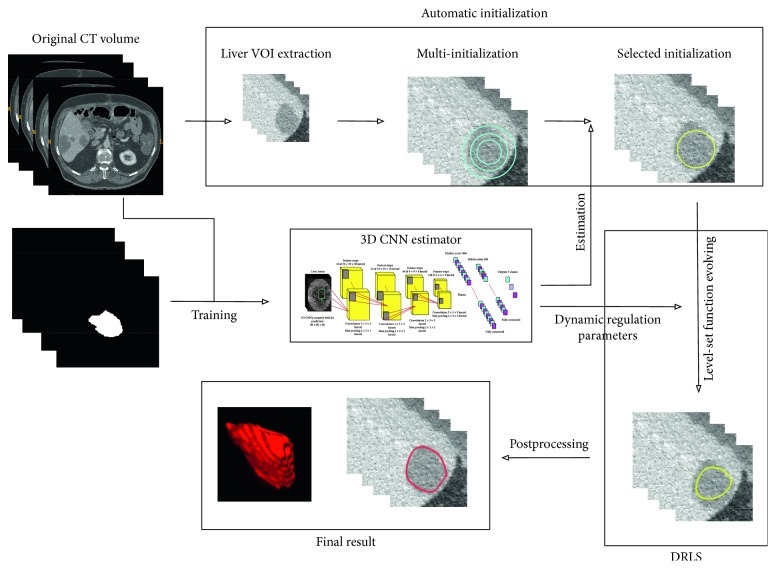
The proposed liver tumor segmentation framework that consists of four modules automatic initialization, dynamic regulation of parameters, 3D level set process, and 3D CNN training and prediction.

Our proposed method is a hybrid combination of the 3D level set and deep learning with 3D convolutional neural network. Entire procedure of the algorithm is interactive with each other. The proposed liver tumor segmentation framework is illustrated in Figure 1, which consists of four major modules: (1) automatic initialization, (2) dynamic regulation of parameters, (3) 3D level set process, and (4) 3D CNN training and prediction. Besides, there is also a required VOI for segmentation, which can be obtained by some effective tumor detection methods [35]. Our proposed 3D CNN model is able to output the position probability of 3D evolving contour relative to the liver tumor boundary. Since a proper initialization helps the level set framework to acquire a more accurate segmentation, our proposed model can identify many candidates which are closer to the boundary. After automatic initialization, as a result of the level set framework with fixed parameters that causes a poor performance, the probabilities are used to update the parameters of the energy functional in the iterative process. Dynamically regulated parameters tend to lead the evolving contour to ground truth in low contrast and noisy texture. The entire process of our proposed DRLS method is automatic, and its components will be discussed as follows.

#### 2.2.1. Dynamic Regulation of Parameters

There are three parameters weighting the level set functional in equation (7). For one thing, *μ* is responsible for the smoothness of the evolving contour. In practice, we usually set *μ*=0.1, and the value of *μ* has few impactions on segmentation performance. For another thing, on the model of the local level set [17], interior and exterior texture features of the local region decide the points' movement. Therefore, the value and ratio of *λ*_1_ and *λ*_2_ play a crucial role in the direction and magnitude of curve evolution over iterations. Therefore, we design a 3D CNN to estimate the location of the zero level set contour relative to the tumor. Outputs of probabilities *p*_1_, *p*_2_, and *p*_3_ express three possible locations: inside, near, or outside the tumor boundary, respectively. By the following equation, we use the 3D CNN outputs value to set the weighting parameters *λ*_1_ and *λ*_2_ of speed equation (7):

(8) $$\begin{array}{c} \lambda_{1}=\exp\left(\frac{1+p_{1}+p_{2}}{1+p_{2}+p_{3}}\right),\\ \lambda_{2}=\exp\left(\frac{1+p_{2}+p_{3}}{1+p_{1}+p_{2}}\right). \end{array}$$

**Figure 2:**
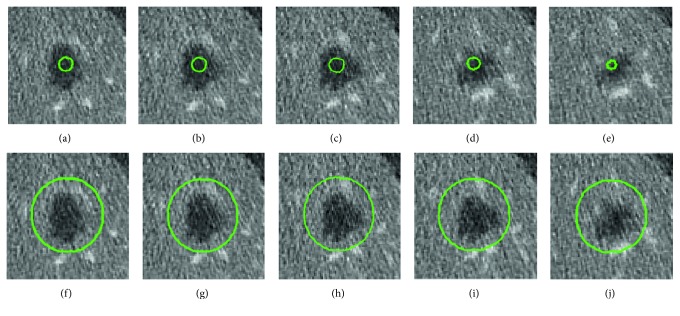
Estimated probabilities of evolving contours position in adjacent slices. (a)–(e) Contour inside the tumor with *p*_1_=0.78, *p*_2_=0.14, and *p*_3_=0.08, displayed by selected adjacent slices in a volume. (f)–(j) Evolving contour outside the tumor boundary *p*_1_=0.1, *p*_2_=0.17, and *p*_3_=0.73. Green circles are the zero level set contour in the 3D grid.

As Lankton and Tannenbaum proved [17], *λ*_1_ and *λ*_2_ actually controls two mutual restricted forces within opposite directions. If *p*_3_ ≫ *p*_1_, then *λ*_2_ > *λ*_1_, and the contour has a tendency to expand. Conversely, if *p*_1_ ≫ *p*_3_, then *λ*_1_ > *λ*_2_, and the contour is more likely to contract. When *p*_2_ ≫ *p*_1_, *p*_3_, it denotes the evolving contour is close to the boundary, and two parameters are similar. The exponential function is used to increase the range of values and ratios that *λ*_2_ and *λ*_1_ can benefit from.  Figure 2 illustrates the predictions to evolve contour positions by our designed 3D CNN model.

#### 2.2.2. Automatic Initializations

Active contour and related curve evolution segmentation methods are dependent on proper initializations. For fully automatic segmentation, we give a self-adaptive solution to search an appropriate initial contour close to the tumor boundary. Before segmentation, several initializations with different radii were provided, for example, 6, 10, 20, 30, and 40 pixels in our work. To every scale of contours, we use the 3D CNN classifier to predict each location probabilities. The one nearest to the tumor boundary with the maximum of *p*_2_ is selected as the ultimate initialized approach.

#### 2.2.3. 3D CNN Architecture

3D CNN produces estimates for the classification of input interesting volume by taking local contextual image information into account. This is achieved by sequential convolutions of the input with multiple filters at the cascaded layers of the network. Each layer *l* ∈ [1, *L*] consists of feature maps produced by a nonlinearity function:

(9) $$\begin{array}{c} y_{l}^{m}=f\left(\overset{C_{l-1}}{\underset{n=1}{\sum }}k_{l}^{m,n}*y_{l-1}^{n}+b_{l}^{m}\right), \end{array}$$

where *C*_*l*_ is referred to as number of channels or feature maps in the *l*_th_ layer. Equation (9) is the result of convolving each of the previous layer's channels with a learned kernel *k*_*l*_^*m*,*n*^, adding a bias *b*_*l*_^*m*^ and applying a nonlinearity *f*. *y*_*l*_ is believed a particular pattern, i.e., a feature, associated with the FMs. These learned features reflect the predominance of the deep learning technique being good at detecting potential and complex rule among the training sets. Appropriate convolutional kernels decide the effect of CNN. Traditional 2D convolution has paid no consideration on adjacent slices relationships, with difficulties to serve three-dimensional data for classification. Therefore, we prefer the 3D convolution kernel to extract features efficiently and predict the 3D evolving contour positions over iterations.

**Figure 3:**
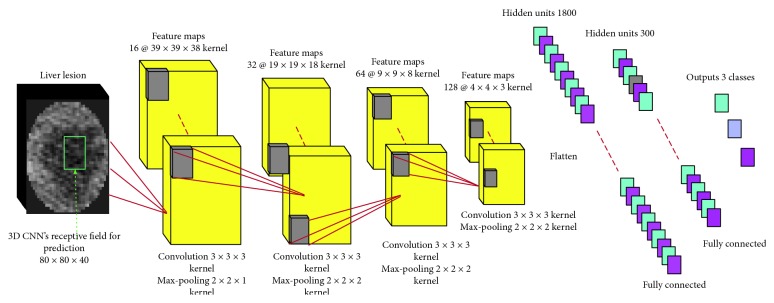
Our baseline 3D CNN consists of four layers with 3^3^ kernels for feature extraction, leading to a receptive field of size 46 × 46 × 23. The classification layer is implemented as convolutional with 2 fully connected layers, which enable dense-inference. When the network is fed an input of 80 × 80 × 40, it predicts three classes simultaneously, one for each shift of its receptive field over the input. Number of FMs, convolutional filters, and their sizes are depicted as number@size, respectively.

The baseline network of the proposed method is depicted in Figure 3. Our architecture consists of four convolutional layers followed by three fully connected layers, including the final three-node layer. All convolutional layers use 3 × 3 × 3 kernels for feature extraction and 2 × 2 × 2 for max pooling except the first layer. Too large 3D kernel size costs massive time, and the smaller does not take accept effects. We set the depths of the convolutional layers 16, 32, 64, and 128, respectively. The depth and filter size can be tuned if necessary, but these parameters were chosen based on examination of highly diverse tumor datasets. In nonlinearity function *f*, each convolutional layer consists of addition filters: batch norm and PReLU. Batch norm is a solution of distribution imbalance in the training set [36], which can learn inherent features and rules of collected data. PReLU is a kind of nonlinear function ReLU:

(10) $$\begin{array}{c} \text{PReLU}\left(x_{i}\right)=\left\{\begin{array}{cc} x_{i}, & \;\text{if}\;\;x_{i}>0,\\ a_{i}x_{i}, & \;\text{if}\;\;x_{i}\leq 0, \end{array}\right. \end{array}$$

where if *a*_*i*_=0, PReLU equals ReLU. If *a*_*i*_ is close to zero like 0.01, PReLU is replaced with leaky ReLU. PReLU has obvious advantages of fast convergence and low error rates, avoiding from early zeroing that causes inactive neurons when training [37]. Max pooling is the final step of the convolutional block. We take a 2 × 2 × 2, except the 1st layer, pixels subregion from the PReLU output representing the region by its maximum value, thus reducing the dimensionality of the dataset.

#### 2.2.4. Training of the 3D CNN

The cost function of 3D CNN is the cross entropy loss, minimizing the log likelihood of the volume input:

(11) $$\begin{array}{c} L_{\text{loss}}\left(T,f\left(\omega ,x\right)\right)=-\frac{1}{N}\overset{N}{\underset{n=1}{\sum }}\overset{M}{\underset{m=1}{\sum }}T_{m,n}\;\log\left(p_{m,n}\right)+\frac{\eta }{2}{\left\|\omega \right\|}^{2}. \end{array}$$

Unlike 2D CNN training, a batch of the training set contains only one individual volume CT images. In equation (11), *T* tells the true label for the example of every batch and *f*(*ω*, *x*) is the prediction via 3D CNN. When the current contour of the 3D level set is located inside the tumor, *T*_*m*,*n*_ equals 0. If near the tumor boundary, the value is 1. Otherwise, *T*_*m*,*n*_ equals 2. *p*_*m*,*n*_ gives the probability of the *n*_th_ example being classified into the *m*_th_ class as the output of *f*(*ω*, *x*). (*η*/2)||*ω*||^2^ penalizes the size of the weights in the model, where the coefficient of regularization selects *η*=0.00004. Stochastic gradient descent with momentum is applied to update the weights (*ωw*_*i*_) of the network [23]:

(12) $$\begin{array}{c} v_{i+1}=\zeta v_{i}-\alpha ▽\omega_{i}-\eta \omega_{i},\\ \omega_{i+1}=\omega_{i}+v_{i+1}, \end{array}$$

where *i* is the iteration step, *v* is the previous gradient, and *η* is the same value of the regularization coefficient that appears in equation (11). *ζ* is initialized to 0.9 for decay. Momentum-based methods damp the gradient and provide better convergence rates for deep networks. For weights of initialization, we use truncated normal distribution to make sure they are within a reasonable range of normal distribution *N*(0, 10^−2^). The learning rate is initialized at 0.01. For every global step (100), the learning rate is adjusted with decay factor 0.1 exponentially. In order to preserve identical distribution of 3D examples, we use batch normalization preventing internal covariate shift when training [36]. The coefficient of decay is same as before and *ε*=0.001. Meanwhile, the rate of drop out is set to 0.7 against overfitting.

#### 2.2.5. Dynamic Regulation of Local Window Size

The segmentation performance of the local region level set model has a close relationship with the local window size of points on the contour. If the size is relatively small, the points cannot be aware of enough areas, which lead to a failure of curve evolution. Instead, overlarge window may cause unexpected segmentation completely. Hoogi et al. presented an algorithm of adaptive local window for the 2D level set [21]. The method considered the object scale, the spatial texture, and the changes of the energy functional over iterations. The values of local window size were calculated with the current gray level cooccurrence matrices (GLCM). The authors had proved its effectiveness compared with traditional ones. However, in the 3D level set, computing of GLCM is too time-consuming and terrible. Moreover, it requires prior knowledge of tumor size in three dimensions which causes a semiautomatic segmentation. Therefore, we propose a lightweight and fully automatic framework to dynamically regulate the local window size based on contour position probabilities, which is calculated using the following equation:

(13) $$\begin{array}{c} \left\{\begin{array}{cc} \text{rad}=8, & \;0.4<\left|p_{2}\right|<0.6,\\ \text{rad}=5\times \left\lceil 5\times \left|p_{1}-p_{3}\right|\right\rceil , & \;\text{otherwise}. \end{array}\right. \end{array}$$

where rad represents the local window size that is updated synchronously over iterations. The condition of 0.4 < |*p*_2_| < 0.6 denotes that when evolving contour is located near the tumor boundary, and 8 pixels is provided that achieved better results on average during testing. Otherwise, the contour has a high possibility inside or outside the tumor. To this case, based on the distance between *p*_1_ and *p*_3_, we give a dynamic value. If the contour is farther away the tumor boundary, rad would be increased relatively, and vice versa. Sufficient window size guarantees the point could move toward target in the right way and converge quickly.

### 2.3. Implementation Details

#### 2.3.1. Reinitialization

The level set function (LSF) often becomes very flat or steep near the zero level set in the evolution process and this will affect the numerical stability. A remedy procedure of reinitialization is applied periodically to enforce the degraded LSF being an signed distance function (SDF). Higher dimensional level set function tends to more unstable numerical, where the reinitialization plays a crucial role. We tried several classical methods that failed to deal with 3D LSF evolution in segmentation. For example, the method proposed by Sussman et al. [38] has difficulties to preserve the shape of SDF in 3D LSF. Giovanni et al. used a true upwind discretization to make the interface localization accurate [39]. Although LSF is kept more stable, it limits possible evolution and prevents new zero contours from emerging. In recent years, the idea of regularizing evolution results in the eliminating reinitialization procedure [12, 40, 41]. These methods have many advantages over traditional ones with higher efficiency and easier implementation, but they can only be applied to some variational LSF in specific forms out of three dimensions. In this work, we apply the reaction-diffusion (RD) method to maintain the numerical stability of the level set without reinitialization [42]. Through experiments, RD had a better performance on the MS model.

#### 2.3.2. Building of Training Sets

**Figure 4:**
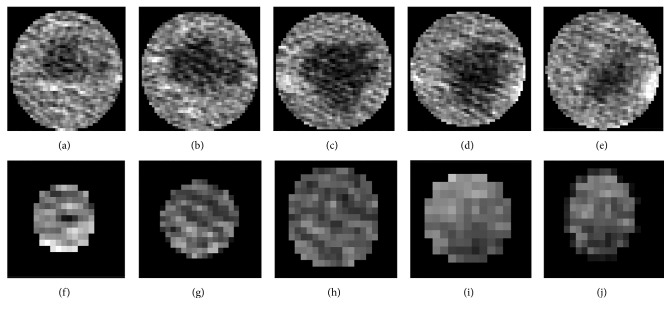
Construction of the training sets in series. The first row (a)–(e) is the set of label “outside,” where the contour is far away from and out of the tumor boundary. The second row (f)–(j) of adjacent slices is labeled as “inside.” Two classes have distinct features for learning.

In our work, the evolving contour is an individual volume which requires prediction of its current position by 3D CNN. To build training sets, we sampled examples of volume located inside the tumor as the “inside” label, which has an obvious distance away the boundaries and got the volumes close to tumor boundary labeled “boundary” class. Similarly, ones that have a larger radii are classified to “outside.” Figure 4 describes classes of the training set in adjacent slices. Because the proposed 3D CNN has fully connected layers, it requires the fixed size of inputs for learning and prediction. The extracted training examples are resampled to 80 × 80 × 40. Although most of tumor's thickness is under 20, for enhancing features, depth of 40 makes a tradeoff between computing efficiency and learning effects.

#### 2.3.3. Data Augmentation

Deep neural network, due to its scale and complexity, typically requires substantial datasets to perform optimally. The proposed 3D CNN is sensitive to directions of the training set, which needs data augmentation and multiorientation pooling in the 3D grid. Therefore, we applied rotation stochastically around an axis by (−45 ≤ *θ* ≤ 45) with an uniform scaling factor (0.8 ≤ *ε* ≤ 1.2). Then, translations (−2 ≤ *x* ≤ 2, −2 ≤ *y* ≤ 2, −1 ≤ *z* ≤ 1) were made on images.

## 3. Results

### 3.1. Data

We trained the proposed 3D CNN model and tested our method on the competitive datasets of MICCAI 2017 LiTS Challenge and 3DIRCADb. The LiTS dataset contains 131 and 70 contrast-enhanced 3D abdominal CT scans for training and testing, respectively. They were acquired by different scanners and protocols from six different clinical sites, with a largely varying in-plane resolution from 0.55 mm to 1.0 mm and slice spacing from 0.45 mm to 6.0 mm. 3DIRCADb includes 15 CT volume images involving 120 liver tumors of different sizes. The pixel spacing, slice thickness, and number of slices varied from 0.56 to 0.87 mm, 1 to 4 mm, and 74 to 260, respectively, with the in-plane resolution of 512 × 512 pixels in all cases. Tumors manually segmented by clinical experts were also provided and considered as ground truth. In our experiment, we exclude tumors with short axis less than 5 pixels, since they are too small and only visible in several slices. In investigation of all cases, tumor size varied widely. Their textures were highly diverse in terms of spatial homogeneity and contrast, which increases the significance of dynamically regularizing parameters in the process of the level set. We used images with the original resolution to avoid possible artifacts from image resampling, employing distinct details for accurate tumor segmentation.

### 3.2. Evaluation Metrics

Our segmentation method was evaluated using six quantitative metrics, including dice similarity coefficient (DICE) [43], volumetric overlap error (VOE), relative volume difference (RVD), average symmetric surface distance (ASD), and average symmetric RMS surface distance (RMSD) [3]. The DICE is used for precise evaluation of the segmentation results, with a higher number indicating a better result. The VOE is the nonoverlapping ratio of the segmentation result and ground truth data. It is also used to evaluate the precision of the results, with a lower number indicating a better result. The RVD is used to evaluate whether the algorithm tends to oversegmentation or undersegmentation. RVD is negative if the algorithm undersegments the object, and 0 is optimal. ASD and RMSD (in millimeters) evaluate the distance of the border voxels of segmentation and ground truth in different measurements. For both of them, a lower value denotes a better segmentation.

### 3.3. Volume of Interest and Initial Distance Map

To evaluate DRLS performance on dynamic regularizing parameters, we selected 40 and 20 tumor volumes from the LiTS dataset with different sizes for training and testing. In testing, for the definition of the volume of interest to the level set process, we invited experienced radiologists to mark the center point on the short axis and extract VOI. The testing dataset had an average short axis of 23.3 ± 6.85 pixels. A bounding box was then generated of size 80 × 80 × 60, which makes sure the entire tumor and its surroundings were included in the segmentation process. To construct distance map *ϕ*(*x*, *y*, *z*), zero level set were initialized using radii of 6, 10, 15, 20, and 30 pixels, respectively. These radii generated initial contours that were balls located inside or outside the tumors, while others were close to the tumor boundaries. Conventional level sets are sensitive to different initializations. The broad range of initializations allowed us to evaluate the strength of our method in dealing with initial contours far away from the tumor in either direction.

### 3.4. Segmentation Performance

**Figure 5:**
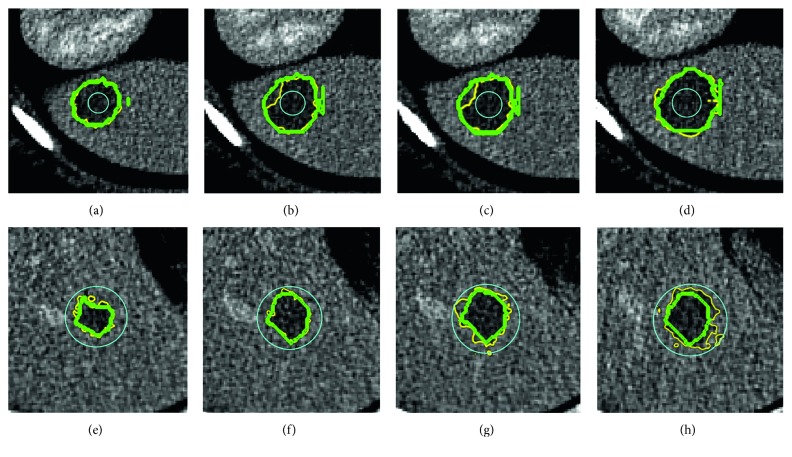
Tumor segmentation using the proposed method DRLS with two different initializations. (a)–(d) Adjacent slice examples—small initialization (6 pixels radius); (e)–(h) large initialization (20 pixels radius). Cyan: initial contour; green: manual radiologists' annotation; yellow: our final segmentation by DRLS.

Traditional 3D level set is sensitive to two things: texture or contrast features of foreground and background and a wide range of optional initial contours. We test 20 3D CT tumor scans with our proposed method dynamic regulation level set (DRLS). For all five different initializations, average DICE and standard deviation of 0.76 ± 0.10 were found on the MS energy model.  Figure 5 shows some examples of adjacent slices in volumes.

### 3.5. Dynamic Regulation Process

**Figure 6:**
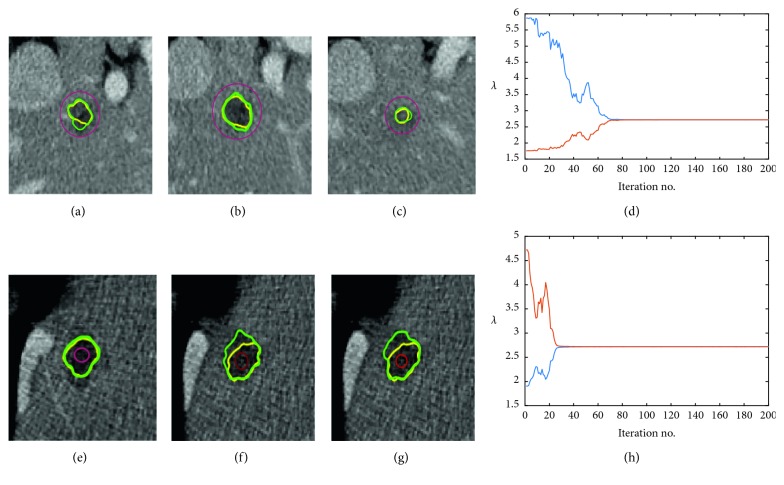
The evolution of parameters *λ*_1_ and *λ*_2_ over iterations of the segmentation process. (a)–(c) Initial contour outside the tumor in adjacent slices of a volume data; (e)–(f) initial contour inside the tumor. Green: manual radiologists' annotation; red: initial contour; yellow: result of our proposed method. (d, h) Statistics of *λ*_1_ and *λ*_2_ over 200 iterations.

As Lankton and Tannenbaum [17] described, parameters of *λ*_1_ and *λ*_2_ control a couple of mutual restrictive inward and outward forces. If inward force weights more heavily, the evolving contour has a stronger tendency to contract, and vice versa. When the current evolving contour has a long distance out of the tumor boundary, a relatively larger *λ*_1_ could help the zero level set move toward the target. In addition, in Lankton's model, each point's force is decided by local region texture. As a result of heterogeneous intensity existing widely in tumor CT images, each local motive force is different, and fixed parameters tend to make level set functional trapped into local minima, providing worse results. Consequently, the dynamic parameters based on the specific texture feature have the potential to resolve this problem. In usual, an initial contour close to the tumor boundary always obtains a better segmentation. In order to evaluate the robustness of our method, we chose two initial contours outside or inside far away the tumor boundaries.  Figure 6 illustrates two examples of the parameters evolution process. At the beginning, *λ*_1_ and *λ*_2_ had a large difference because of an explicit contour position classification, with a strong motional tendency. When close to boundaries, similar *λ*_1_ and *λ*_2_ were similar and local textures decided the final result.

### 3.6. Energy and Dice Convergence

**Figure 7:**
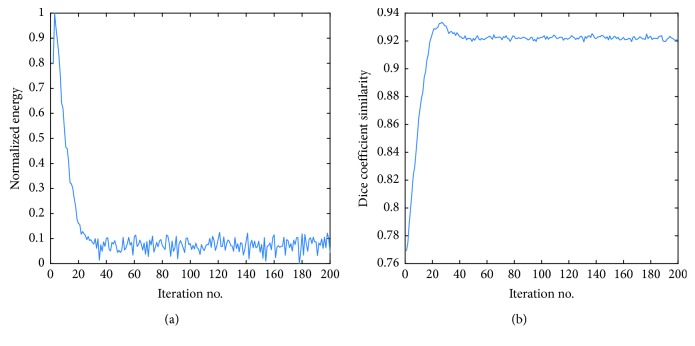
(a) The statistics of energy functional and (b) dice similarity coefficient evaluations over 200 iterations.

Figure 7 demonstrates the convergence of the DICE and energy functional over 200 iterations for one case with our proposed method. In Figure 7(a), after a short part of increasing, the energy decreased with increasing iterations, converging on a very narrow interval. In the right one, DICE increased rapidly when the automated segmentation converged on a result that matched the manual segmentation. On different texture features, as expected, both metrics obtained substantial convergence after 40 steps and there were only minor fluctuations around their final values over later iterations.

### 3.7. Comparison with Fixed Parameters Level Set

**Figure 8:**
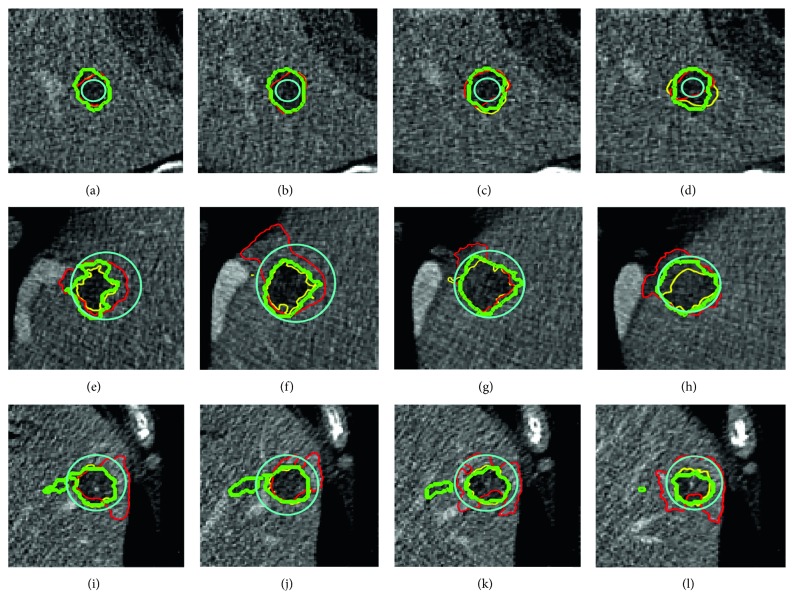
3D liver tumor segmentation using DRLS and FLS in comparison. (a)–(d), (e)–(h), and (i)–(l) are adjacent slices of three 3D CT scans. (a)–(d) are located inside the tumor, and the other two are close to or outside the tumor boundary. Cyan: initial contour; green: manual radiologists' annotation; red: FLS result; yellow: our proposed method.

**Figure 9:**
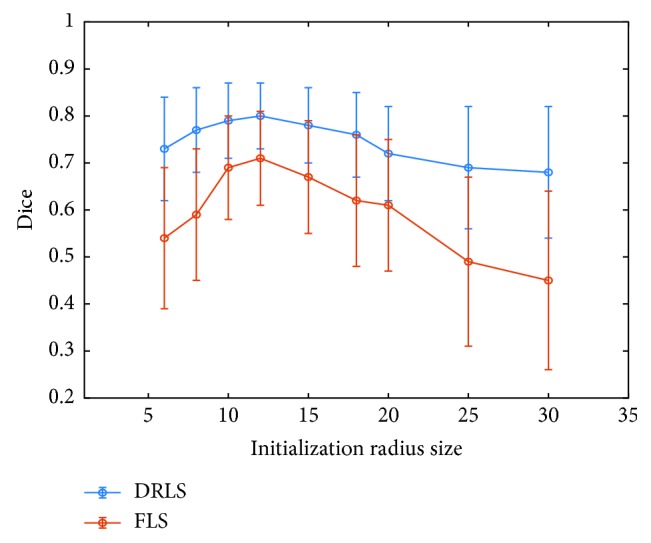
Mean ± SD of dice coefficients over the whole testing datasets with different initializations for MS energy.

**Figure 10:**
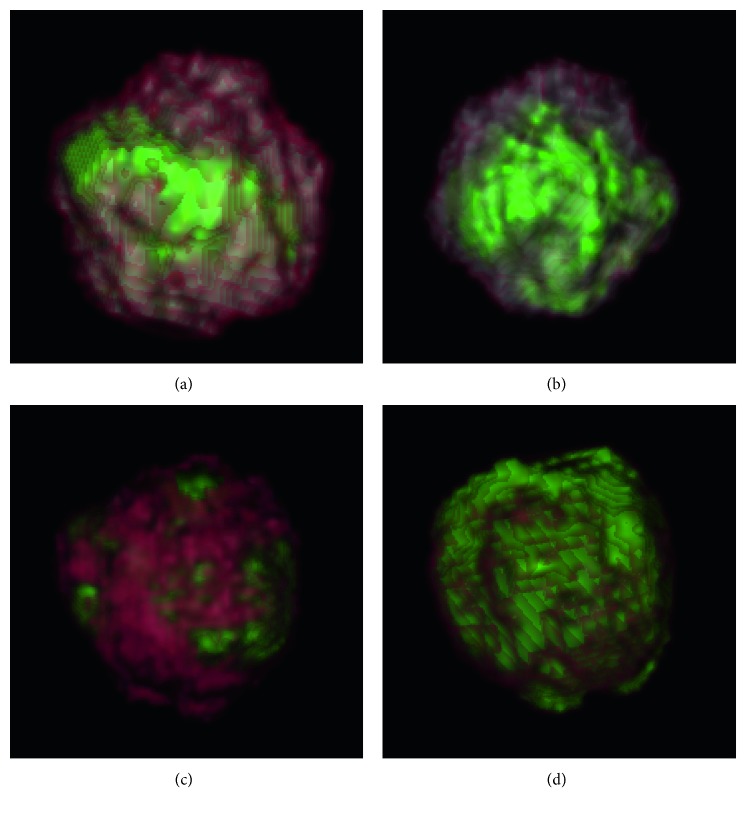
Segmentation results obtained by the FLS and our proposed method DRLS. Each row shows one case of tumor segmentation results in 3D reconstruction. The left column of tumors (a, c) is processed with FLS and the right (b, d) with DRLS. The surface of the ground truth is in green. The surface of the segmented tumor by the corresponding method is in red.

We compared our method (DRLS) with a state-of-the-art local framework of level set segmentation [17], which uses a pair of fixed parameters *λ*_1_, *λ*_2_, and a fixed local window size (FLS) during the process of segmentation. To determine the fixed parameter, we tested several values and *λ*_1_=*λ*_2_=1 were chosen. In addition, we preferred local window size of 10 pixels for points on the contour. Those fixed parameters values were selected for FLS since they supplied the average best performance for 20 testing cases.  Figure 8 demonstrates different tumor characteristics, initial contour sizes, and final segmentations, using DRLS and FPLS methods. The first row of adjacent slices shows an approximate result with the same initial contour radius 6 pixels, where DRLS is better than FLS obviously. The second and third rows are instances that reflect our methods' great advantages. The tumors are close to the boundaries of the liver, by which evolving contours tend to be attracted easily using FLS. In contrast, our proposed algorithm regulates the control parameters in time and drives contours to the correct direction. For quantitative analysis, we evaluated DRLS and FLS with 6, 10, 15, 20, and 30 pixels radii of initializations. To have a comprehensive comparison, we listed the reported tumor segmentations results on the testing dataset. Results in Table 1 show that our proposed method achieved better performance consistently in five initializations. DRLS had average DICE of 0.76 ± 0.10 for the MS energy model, and FLS had 0.61 ± 0.16, respectively. To other metrics, DRLS holds significantly lower error values proven to be close to the ground truth. To verify influences of different initial contours between DRLS and FLS, we increased DICE evaluations on more radius sizes (6, 8, 10, 12, 15, 18, 20, 25 and 30 pixels). Results are illustrated in Figure 9. Since the testing dataset had an average short axis of 23.3 ± 6.85 pixels, the initial contours with 10 and 12 pixels radii that are closer to the tumor boundary acquired better DICE for DRLS and FLS. Besides, it is obviously found that DRLS provides more stable performance which is less sensitive to different initializations.  Figure 10 illustrates some examples of 3D reconstruction in comparison of two methods.

### 3.8. Comparison to Other State-of-the-Art Methods on 3DIRCADb

To validate the effectiveness and robustness of our method, we also conducted experiments on the 3DIRCADb dataset, which is publicly available and offers a higher variety and complexity of livers and tumors. In experiments, we divided the 3DIRCADb testing dataset into two categories. A tumor with longest axis larger than or equal to 40 pixels was defined as a large one, and a small tumor, vice versa. Initialization of DRLS and its local window size was automatically adjusted by contour location probabilities. As shown in Table 2, our method achieved an average VOE and RVD of 26.93% and 6.55%, an average ASD and RMSD of 1.05 and 1.44 mm, and an average DICE of 0.85, respectively. For large tumors, a VOE of 22.87%, RVD of 3.29%, ASD of 1.09 mm, RMSD of 1.52 mm, and DICE of 0.87 were provided. For small tumors, a higher VOE of 33.29% and a lower DICE of 0.81% were obtained. From the comparison, it claimed that the performance gain is mainly attributed to the improvement of the large-scale tumor segmentation results. This is mainly because that the 3D CNN classifier is more effective when the receptive field has more texture information. Once its output probabilities are accurate, the dynamic parameters are estimated properly so that the large 3D tumor can be improved by a large margin. Although the hybrid method still benefits small tumors, the effect is limited since such size contains a few features which lead to an unstable prediction.

Table 3 shows the quantitative segmentation results compared with other state-of-the-art algorithms on the 3DIRCADb dataset. We can see that our method achieved the better performance on DICE than [29–33, 44–47] on liver tumor segmentation. In this experiment, we collected parts of the 2017 LiTS dataset to train the 3D CNN classifier and used the well-trained model to test DRLS on the 3DIRCADb dataset directly. The approach of selecting initialization and local window size adopted the strategy of initial contour probabilities. The evaluation metrics involved VOE, RVD, ASD, RMSD, and DICE.

Han [29] presented a model derived from U-Net, which combined the ResNet model in a number of layers. Similarly, Chlebus et al. [44] proposed another neural network model for segmentation with fuzzy clustering based on random forest for postprocessing. The backbone of the entire network depended on the 2D U-Net baseline. Both of models [29, 44] are 2D networks with fully automatic detection for tumors, which pay little attention to features between adjacent slices, providing lower VOEs and DICEs. Moghbel et al. gave a fully automatic segmentation without any interactions, integrating cuckoo optimization and fuzzy c-means with random walkers. The performance of our method on the 3DIRCADb dataset was comparable to that of Moghbel, giving 0.1 DICE improvement on average. In addition, Foruzan and Chen [31] and Wu et al. [32] designed semiautomatic segmentation based on topological methods like watershed and graph cut. Because of requiring prior knowledge, these semiautomatic algorithms acquired good results with DICE 0.82 and 0.83, respectively, when VOE or other metrics dropped dramatically. Actually, although semiautomatic ones cannot be compared directly with fully automatic ones, to some extent, their reported results on the 3DIRCADb dataset can give expression to the state-of-the-art performance for the tumor segmentation task. Christ et al. [30] used cascaded fully convolutional neural networks enabling the segmentation for low-contrast CT images. The first pipeline extracted the liver as the ROI input for the tumor segmentation via second FCN. As a result of the limited sole FCN's ability, this model took DICE of 0.56 only. Huang et al. [33] also required an accurate initial eclipse, enclosing the middle slice of 3D tumor scans. Living on desired initialization, it provided an average of 27.05 VOE and 0.84 DICE. Zheng et al. [46] proposed a level set framework combining DRLSE [40] and HMRF-EM constraint [48] to estimate the evolving contour with fixed parameters, which served a higher VOE, RVD, ASD, and RMSD. Jin et al. [47] set up a multitasks 3D model for tumor location and segmentation. The part of network backbone for fully automatic segmentation was derived from 3D U-Net with residual block holding a mean DICE score of 0.83. In the experiment, we collected parts of the LiTS dataset as the training dataset for the proposed 3D CNN model. As shown in Table 3, our method achieved DICE 0.85 ± 0.06 that had a clear advantage on the 3DIRCADb dataset, when VOE, RVD, ASD, and RMSD had a stable performance in all cases.

**Figure 11:**
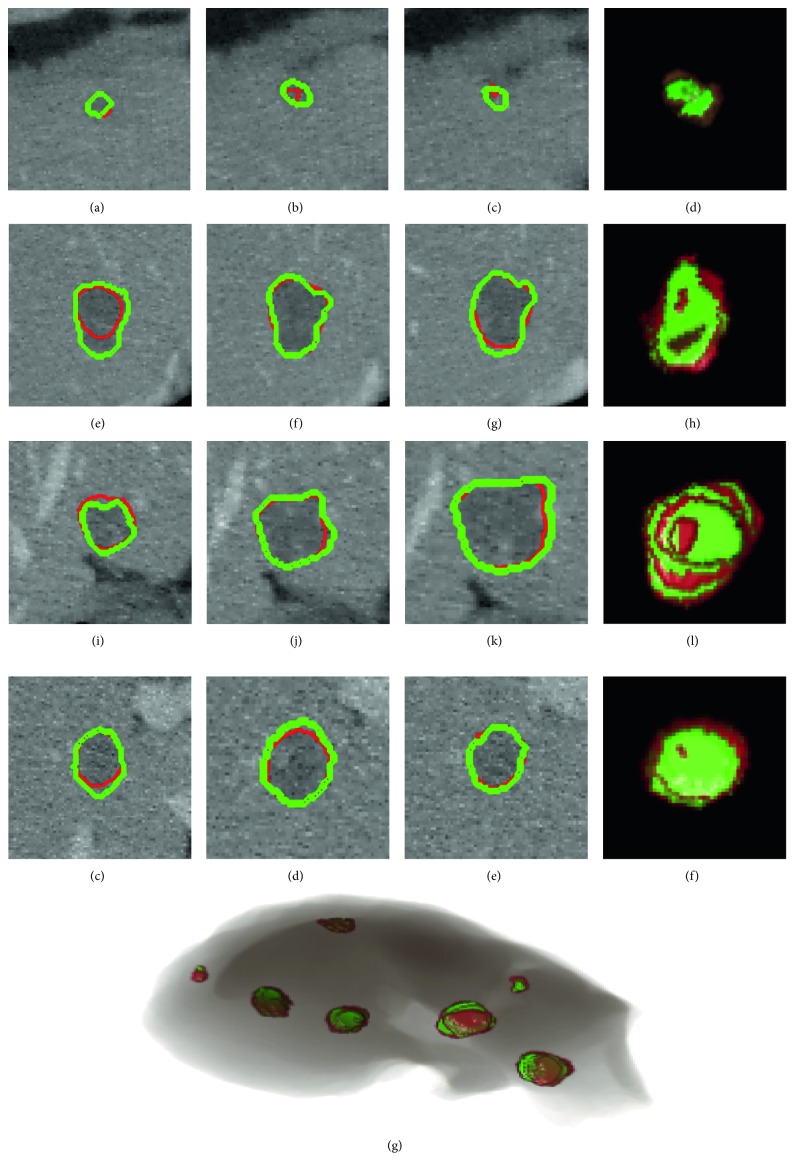
Segmentation results obtained by the proposed method on the 3DIRCADb dataset. (a)–(p) One case of tumor segmentation results in adjacent slices and the 3D reconstruction result. (q) 3D reconstruction result of the liver and its entire tumors are inside. Green is the ground truth annotated by radiologists. Red is the segmentation result by DRLS.

The promising results indicated the effectiveness and good generalization capability of our method. In other words, such a good result was benefited from the LiTS dataset, which contains a great number of cases with large variations in features of tumor. It is the diversity that devotes the 3D CNN model to extract discriminative features from training set more effectively.  Figure 11 shows some typical examples with different sizes, intensities and positions. It can be seen that our method achieved comparable results to the ground truth manual segmentation in most cases. However, relatively large undersegmentation and oversegmentation errors occurred when the tumor presented ambiguous or low contrast boundaries.

## 4. Discussion

For accurate and robust liver tumor segmentation for clinical diagnosis, we propose a novel method for dynamic regulation of the *λ*_1_ and *λ*_2_ contour parameters in the level set model. Meanwhile, automatic initialization and local window size could be adjusted based on the estimation of the contour position. Depending on a proper VOI, we provide a fully automatic and self-adaptive segmentation method for low-contrast CT scans. The main advantages of the proposed approach are demonstrated: (1) we introduce the 3D CNN to estimate 3D evolving contour's position effectively. The method uses position probabilities to calculate level set parameters *λ*_1_, *λ*_2_, accurate initialization, and local window size, respectively. This process can be generalized for segmentation with active contour methods. (2) Adaptiveness to highly diverse tumor dataset. (3) Less sensitive to accurate initialization, manual interaction, and parameter fine tuning. (4) Superior performance with high robustness to complicated conditions like various tumor's location, shape, and textures, compared with the state-of-the-art methods.

In addition, the whole baseline of our proposed DRLS is automatic. At the beginning, the well-trained 3D CNN model can evaluate the most suitable initial contour from many candidates. In the iterative process, the fixed parameters of the level set are regulated dynamically based on the position probability of 3D evolving contour until energy convergence. This novel automatic framework combining hybrid methods provides more accurate liver tumor segmentation especially in low contrast and noisy CT scans. To show the generalization capability of our method in the clinical practice, we tested our trained model on the MS energy functional and a wide range of sizes and locations of 3D initial contours. We compared our proposed method DRLS with the state-of-the-art level set frameworks and several methods based on deep learning.  Figure 8 illustrates that our algorithm DRLS method can deal with different types of 3D tumor scans with low contrast, heterogeneous, noisy background, or close to liver edge, outperforming commonly used level set framework with predefined fixed contour parameters FLS. As proven, Table 1 and Figure 9 demonstrate that DRLS has more accuracy and robustness regardless of the initial contour location and size, holding an average dice 0.76 higher than FLS 0.61 on MS energy. Besides, other metrics like VOE, ARVD, ASD, and RMSD had an obvious decline. Our proposed classifier based on 3D CNN is trained by a part of the 3DIRCADb dataset. Furthermore, we tried DRLS to compete with seven popular semi- or fully automatic segmentation methods on the 3DIRCADb dataset, and it achieved the state-of-the-art results on liver tumor segmentation, with 0.85 DICE on average. These findings indicate two key points. At first, common methods based on active contour cannot provide accurate segmentations of such highly diverse tumor datasets with fixed parameters since different local image features requires different model constraints. Another point is that the hybrid method of deep learning and level set framework can maximize their strengths, detecting region characteristics and delineating details of tumor boundaries. Each tumor type needs completely different sets of energy functional parameters. Using a fixed set of parameters will result in an inaccurate segmentation of the tumor. On the contrary, the energy functional always falls into local minima in the noisy texture. Consequently, our proposed method that combines the level set and 3D CNN model overcomes their respective limitations, serving a significantly advanced result than either method alone.

The presented work has some limitations. First, VOI for each tumor is required and may lead to more accurate segmentation. In the future, we should incorporate the automatic tumor detection at the beginning of the segmentation, so that the entire process will be fully automated and independent out of user's any input. Another future work may include a cascaded joint network integrating multimodalities into a single baseline, exerting each specific advantage. At present, the processes of the level set and deep learning network are mutually independent. Another possible extension is to merger level set framework into neural network as convolutional layers, which implements an end-to-end training system for tumor segmentation.

## 5. Conclusions

We present a hybrid method using the level set and 3D neural network for liver tumor segmentation from CT scans. 3D CNN is used to estimate the current contour position outputting probabilities inside, outside, or close to tumor boundaries. Based on these values, the parameters of the energy functional can be regularized dynamically in each iterative step. Meanwhile, accurate initialization and local window size also can be calculated to improve the performance of automatic segmentation. Compared with the level set framework with fixed parameters, our proposed method (DRLS) archived better results and showed advanced robustness with different initializations. DRLS has generalized a common level set, giving great potential to related research work. To further evaluate our proposed automatic segmentation scheme, we tested DRLS with the well-trained 3D CNN model on the dataset 3DIRCADb. After extensive experiments, the competitive result of our proposed method DRLS was found especially in the large-scale tumor. Some limitations about DRLS will be future work to be optimized.

## Figures and Tables

**Table 1:** Comparison of tumor segmentation results on testing datasets of 20 3D CT scans with five initializations.

Method with init	VOE (%)	RVD (%)	ASD (mm)	RMSD (mm)	DICE
DRLS 6px	35.71 ± 11.94	−6.13 ± 18.58	1.98 ± 1.09	2.21 ± 1.45	0.73 ± 0.11
FLS 6px	52.80 ± 16.22	−19.2 ± 24.95	3.11 ± 1.57	3.95 ± 1.85	0.54 ± 0.15
DRLS 10px	29.73 ± 10.85	0.91 ± 12.81	1.55 ± 1.19	1.81 ± 1.08	0.79 ± 0.08
FLS 10px	41.37 ± 12.80	−4.20 ± 20.14	2.51 ± 1.17	2.85 ± 1.30	0.69 ± 0.11
DRLS 15px	31.35 ± 11.43	2.25 ± 14.08	1.49 ± 1.03	1.87 ± 1.17	0.78 ± 0.08
FLS 15px	44.83 ± 13.19	5.17 ± 19.33	2.66 ± 1.28	3.03 ± 1.42	0.67 ± 0.12
DRLS 20px	36.20 ± 13.38	10.26 ± 19.97	2.01 ± 1.25	2.41 ± 1.33	0.72 ± 0.10
FLS 20px	47.03 ± 16.98	28.60 ± 24.62	2.89 ± 1.34	3.30 ± 1.62	0.61 ± 0.14
DRLS 30px	42.91 ± 16.19	19.83 ± 22.34	2.48 ± 1.46	2.89 ± 1.56	0.68 ± 0.14
FLS 30px	59.72 ± 20.63	43.65 ± 33.72	3.91 ± 1.72	4.46 ± 2.13	0.45 ± 0.19

Results are represented as mean ± standard deviation.

**Table 2:** Quantitative evaluation of the proposed method DRLS on large or small 3D tumor CT scans.

	VOE (%)	RVD (%)	ASD (mm)	RMSD (mm)	DICE
Large tumor	22.87 ± 7.25	3.29 ± 9.92	1.09 ± 0.76	1.52 ± 1.21	0.87 ± 0.06
Max.	35.18	16.83	2.26	4.68	0.96
Min.	7.54	−11.50	0.31	0.29	0.70
Small tumor	33.29 ± 7.99	11.60 ± 21.33	0.91 ± 0.58	1.30 ± 0.99	0.81 ± 0.05
Max.	73.8	49.18	3.27	4.81	0.84
Min.	22.95	−13.75	0.31	0.27	0.48
Average	26.93 ± 8.51	6.55 ± 14.91	1.05 ± 0.71	1.44 ± 1.13	0.85 ± 0.06

Results are represented as mean ± standard deviation.

**Table 3:** Quantitative segmentation results of our method compared to the other novel algorithm on the 3DIRCADb dataset.

Model	Year	VOE (%)	RVD (%)	ASD (mm)	RMSD (mm)	DICE
Moghbel et al. [45]	2016	22.78 ± 12.15	8.59 ± 18.78	—	—	0.75 ± 0.15
Foruzan and Chen [31]	2016	30.61 ± 10.44	15.97 ± 12.04	4.18 ± 9.60	5.09 ± 10.71	0.82 ± 0.07
ResNet [29]	2017	56.47 ± 13.62	—	6.36 ± 3.77	11.69 ± 7.60	0.60 ± 0.12
Christ et al. [30]	2017	—	—	—	—	0.56 ± 0.26
Wu et al. [32]	2017	29.04 ± 8.16	−2.20 ± 15.88	0.72 ± 0.33	1.10 ± 0.49	0.83 ± 0.06
Chlebus et al. [44]	2018	62.55 ± 22.36	−	11.11 ± 12.02	16.71 ± 13.81	0.51 ± 0.25
Huang et al. [33]	2018	27.05 ± 9.19	4.23 ± 19.28	1.49 ± 1.29	2.03 ± 1.91	0.84 ± 0.07
Zheng et al. [46]	2018	28.2 ± 11.9	−8.5 ± 18.5	1.8 ± 1.3	2.4 ± 1.7	—
Jin et al. [47]	2018	—	—	—	—	0.83
Ours		26.93 ± 8.51	6.55 ± 14.91	1.05 ± 0.71	1.44 ± 1.13	0.85 ± 0.06

Results are represented as mean ± standard deviation.

## Data Availability

MICCAI 2017 LiTS Challenge dataset is shared at https://competitions.codalab.org/competitions/17094#learn_the_details. 3DIRCADb dataset is publically available at http://www.ircad.fr/research/3dircadb.
